# Secretory carcinoma of the breast, commonly exhibits the features of low grade, triple negative breast carcinoma- A Case report with updated review of literature

**DOI:** 10.4322/acr.2020.227

**Published:** 2020-12-08

**Authors:** Nirmalya Banerjee, Devmalya Banerjee, Neha Choudhary

**Affiliations:** 1 Postgraduate Institute of Medical Education and Research, Department of Histopathology, Chandigarh, India; 2 Faculty Department of Oncopathology, NH super-specialty Hospital Howrah, West Bengal, India; 3 Faculty Department of Oncosurgery, NH super-specialty Howrah, West Bengal, India

**Keywords:** Breast neoplasm, Secretory component, Sentinel lymph node biopsy, Translocation, genetic

## Abstract

Secretory carcinoma of the breast (SBC) is a rare breast neoplasm. Most of the patients present at an early stage with a relatively indolent clinical course. Lymph node and distant metastasis are also very infrequent. The histomorphological features of the secretory breast carcinoma are quite characteristic. Predominantly three histological patterns, solid, microcystic, and tubular, have been noted with copious amounts of intra and extracellular secretory material. Most commonly, no positivity for estrogen receptor (ER), progesterone receptor (PR) and ERBB2(HER2/neu) is observed in SBCs. As SBC can occasionally be hormone receptor-positive, they should not be categorized in the triple-negative breast carcinoma (TNBC) group in general. A very characteristic genetic translocation t (12;15) has been noted in this rare tumor, resulting in a fusion between ETV6 and NTRK3 proteins. We present a case of a 60-year-old lady who presented with right breast lump of 1-month duration and was managed by lumpectomy and sentinel lymph node dissection. Axillary dissection was not performed because the sentinel lymph node biopsy was negative. Postoperative radiotherapy was given to the right breast with a boost to the tumor bed. No adjuvant chemotherapy was given No recurrence has been noted even after a year of the completion of treatment

## INTRODUCTION

Secretory carcinoma of the breast (SBC) is a rare breast neoplasm. The overall incidence is less than 0.15% and was first described historically by Mc Divitt and Stewart^1^ in the year 1966, and they named it as juvenile breast carcinoma owing to the increased number of cases in the pediatric age group. However, it has been renamed after recognizing that this tumor may occur in a wide age range and also because of its characteristic histomorphological findings.^1^ ^,^ ^2^

Most of the patients present at an early stage with a relatively indolent clinical course. Lymph node metastasis is seen in 30% of individuals, and distant metastasis is also not frequent.^2^ ^,^ ^3^ The histomorphological features of the SBCs are quite characteristic and can be easily distinguishable from invasive duct carcinomas. Predominantly three histological patterns, (i) solid, (ii) microcystic, and (iii) tubular, have been noted with copious amounts of intra and extracellular secretory material. Cellular atypia is usually minimal.^4^ Hormone receptor status is also very much characteristic in SBC. Most frequently, they do not show positivity for estrogen receptor (ER), progesterone receptor (PR) and ERBB2(HER2/neu). Therefore, SBCs very frequently show features of triple-negative breast carcinoma (TNBC*).* They also express cytokeratin 5/6, 14, 17, and c-Kit (CD117), which are also seen in the basal-like breast carcinomas (BLBC).^5^ Typically, 75–80% of the tumors classified as TNBCs belong to the basal-like breast cancer group, and they behave more aggressively and cause rapid vascular invasion.^6^ Transcriptomic analysis of breast malignancy revealed some distinctive genetic signatures, which is the basis for the intrinsic molecular subtyping of breast cancer. BLBC is one of the subtypes. It is defined by the expression of genes characteristic of the basally located epithelial layer of the mammary gland.^7^ BLBCs also uniformly lack molecular targets, which determine the responsiveness of highly effective targeted therapy.^7^ ^,^ ^8^ BLBCs share some features with SBCs in terms of expression of cytokeratin 5/6, 14, 17, and c-Kit (CD117).^5^ A recent study also highlights the heterogeneity within the TNBC group, both morphologically and prognostically. They also encompass certain tumors with a far better prognosis than the other members of this group.^9^ A very characteristic genetic translocation t (12;15) has been noted in this rare tumor, resulting in a fusion between E26 transformation-specific translocation variant 6 (ETV6) and neurotrophic receptor tyrosine kinase 3 (NTRK3) which creates a fusion protein ETV6-NTRK3 resulting in activation of two oncogenic effector pathways.^10^

We present a case of a 60-year-old lady who presented with right breast lump of 6 months duration and was managed by lumpectomy and sentinel lymph node dissection and adjuvant radiotherapy. The radiological, histomorphological, and molecular features of SBC are also discussed.

## CASE REPORT

**Figure 1:**
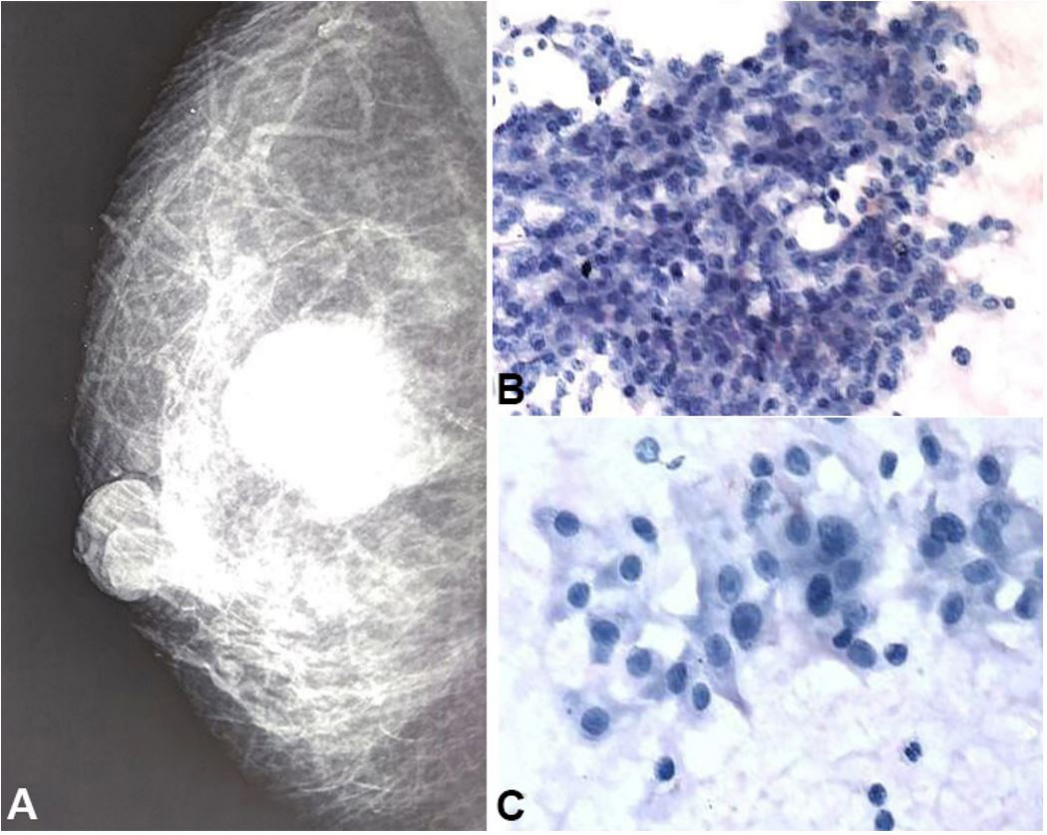
Mammography and photomicrographs of the aspiration smears. A – Well defined, sub-areolar, radio-opaque lesion within the breast; B – Cohesive clusters of tumor cells (Giemsa, 100X); C – Mildly pleomorphic polygonal tumor cells with centrally placed nuclei in a fluid background (Giemsa, 400X).

A 60-year-old lady presented in the surgical outpatient clinic with the chief complaint of a palpable right breast lump over the last month. She had a fine needle aspiration cytology done at a local place, which showed malignancy. On examination, the mass appeared non-tender, firm, mobile, and was located in the central quadrant (Figure 1A). The lesion measured approximately 3x3 cm. No axillary lymph node was palpable at that time. The mammography showed a well-defined, sub-areolar, 3x2 cm radio-opaque lesion with no evidence of microcalcification. Two Giemsa stained aspiration smears undertaken during the consultation were also examined simultaneously. The smears were very cellular, with cells showing minimal nuclear atypia and arranged in cohesive clusters and sheets in a fluid background. No myoepithelial cells were detected. Based on those findings, the retro areolar mass was diagnosed as a malignant epithelial tumor (Figure 11C**)**.

The patient was counseled about the disease status and the need for surgical intervention. A plan of lumpectomy with sentinel lymph node dissection was decided. The sentinel lymph node biopsy was performed, and the tissue was sent to the department of histopathology for an intraoperative frozen section. Grossly, a total of seven lymph nodes was identified in the whole sentinel lymph node specimen, and none of them showed metastatic deposit under microscopic examination. As the sentinel lymph nodes were free from metastasis, no axillary dissection was performed, and only the lumpectomy specimen was sent for final histopathological evaluation.

## Gross and microscopic features

**Figure 2:**
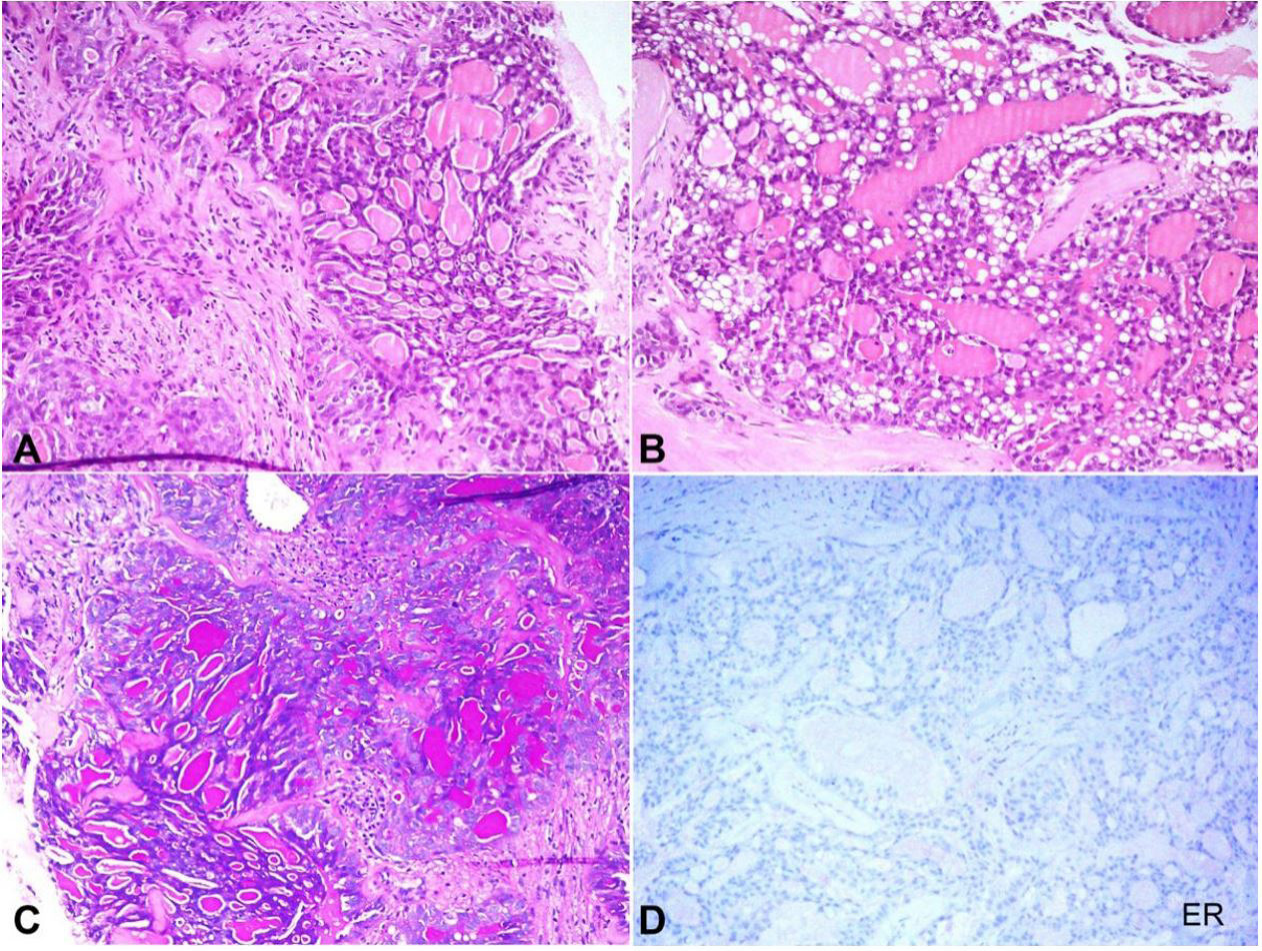
Photomicrography of the tumor. A – Microcystic and follicular arrangement of the tumor cells with eosinophilic extracellular secretion (H&E, 100X); B – Intracellular vacuolation and eosinophilic secretion (H&E, 200X); C – Magenta colored secretion for Periodic acid Schiff stain (H&E, 100X); D – No positivity for ER immunostain (200X).

The lumpectomy specimen measured 2.3x2.2 cm and was oriented with sutures. Adequate sections were taken and were examined under light microscopy. The tumor cells were arranged in tubules, micro cysts, and follicles. A focally solid growth pattern was also noted. Within the micro cysts, there were abundant, pink, eosinophilic secretions, which were intensely positive for Periodic acid Schiff (PAS) and also resistant to diastase. Intracytoplasmic vacuolation and eosinophilic secretions were also noted. Tumor cells were mitotically not very active, and nuclear pleomorphism was also not conspicuous (Figures 222C**)**. All the resection margins were tumor-free (at least more than 10 mm away from the tumor). As a part of the routine evaluation of the breast specimen, hormone receptor study was also performed. Tumor cells were uniformly negative for estrogen (ER) and progesterone (PR) receptors. Evaluation for HER2/neu was done by immunostain and incomplete; faint membranous staining was noted in more than 10% of the tumor cells (Figures 2 D, 33B**).** Hence, the tumor was labeled as negative (score 1) and fluorescent in-situ hybridization (FISH) was not performed.^11^ In addition to that, diffuse membranous and cytoplasmic positivity for cytokeratin 5/6 and S100-P were also documented in the index case (Figures 33D). Hence, we were dealing with a case of secretory breast carcinoma.

**Figure 3:**
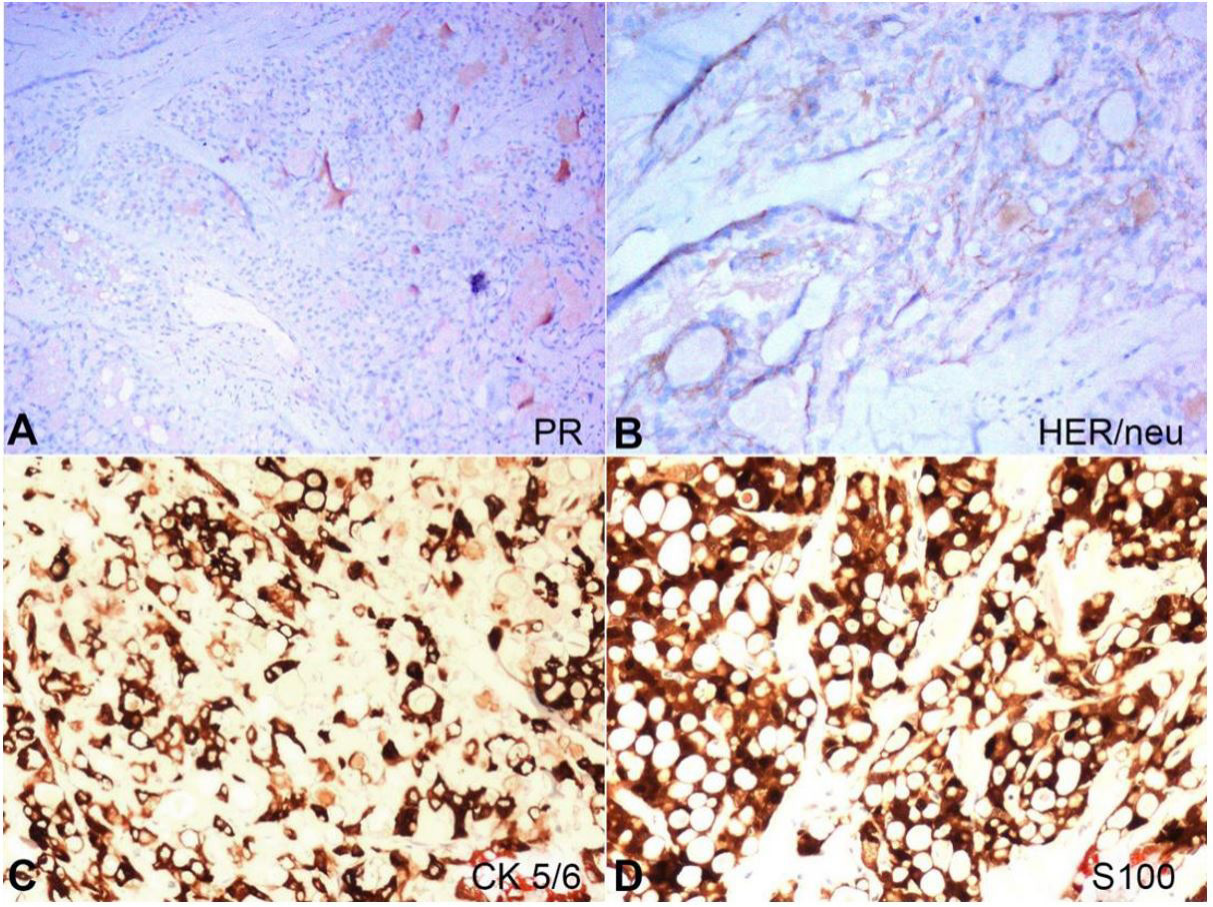
Photomicrography of the tumor: A – No reactivity to PR (100X), B – negative reaction to HER2/neu (200X); C – Diffuse membranous and cytoplasmic positivity for cytokeratin 5/6 (400X); D – Diffuse membranous and cytoplasmic positivity for S100-P (400X).

The FISH study was also performed from the paraffin-embedded sections to confirm the diagnosis. Dual-color break-apart probes (SureFISH^®^, Agilent, St.Clara, USA) were used for FISH evaluation of both ETV6 (12p13.2) and NTRK3 (15q25.3) gene. Fifty nuclei were counted, and red-green split signals were noted in 20 and 19 nuclei for ETV6 and NTRK3 gene, respectively (Table 1, Figure 44B). Therefore, the characteristic translocation was present in the index case. Subsequently, a diagnosis of secretory breast carcinoma was offered in this case. The patient has been kept in a close follow-up. No recurrence or metastasis was noted after a year of lumpectomy, which has been confirmed by a positron emission tomography (PET) scan.

**Table 1:** Result of ETV6 and NTRK3 translocation analysis.

ETV6 translocation	ETV6 (Orange/Green)	5’ ETV6 (Orange)	3’ ETV6 (Orange)	No. of cells
Signals in cell	1	1	1	20/50
NTRK3 translocation	NTRK3 (Orange/green)	5’NTRK3 (Orange)	3’ NTRK3 (Orange)	No. of cells
Signal/s in cell	1	1	1	19/50

**Figure 4:**
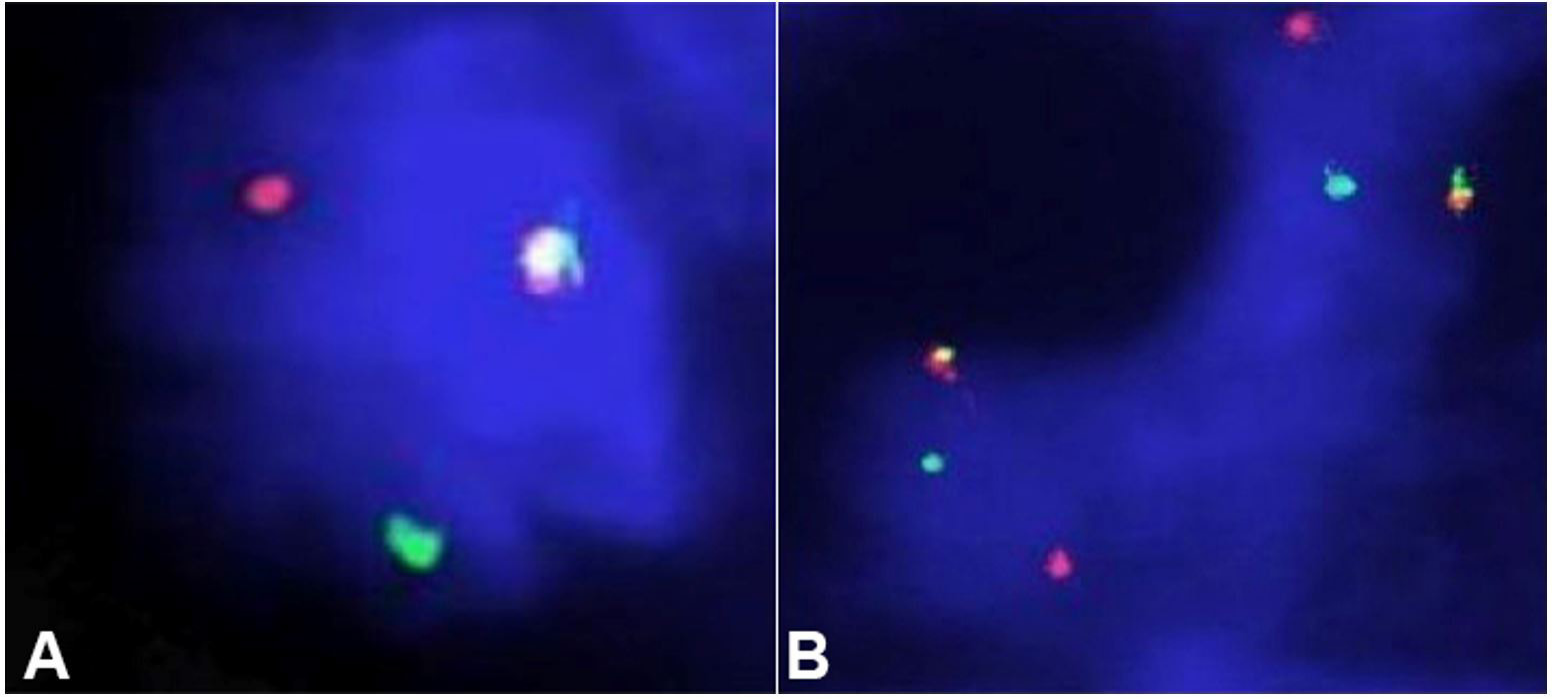
Photomicrography of FISH analysis. A – FISH analysis by dual-color break apart ETV6 probe. One fusion signal (red/green/yellow; arrowed) and one split (rearrangement) signal in the nucleus (400X); B – FISH analysis by dual-color break apart NTRK3 probe. Two fusion signal (red/green/yellow; arrowed) and two splits (rearrangement) signal in the nucleus (100X).

## DISCUSSION

SBC is a rare breast malignancy with an overall incidence of less than 0.15%. It was first reported as juvenile breast carcinoma.^1^ Afterward, it was shown that this tumor affects all age groups and can be detected in both males and females.^12^ According to Jacob et al.^13^ the mean age of SBC is 56 years.^13^ This tumor has an indolent clinical course, and according to Horowitz et al.^2^ the 5-year and 10-year cause-specific survival are 94.4% and 91.4%, respectively.^14^ The location of the tumor varies according to the age group. Adult patients present with upper outer quadrant mass, whereas in the young population, this is most commonly seen in the subareolar region. In the present case also, the tumor was located in the subareolar region which can be explained by the smaller relative size of the breast mound in younger patients deep to the nipple-areola complex.^15^ ^-^ ^17^

Mammographic findings of SBC closely mimic a fibroadenoma as this carcinoma appears as a solitary, discrete lesion with a well-delineated margin. Since this carcinoma is more common in young girls, mammography always has diagnostic limitations because of the denser and glandular elements in the young breast. Ultrasonography of SBC also mimics a fibroadenoma. Mostly a well-circumscribed, hypo to isoechoic mass with micro lobulation is noted on ultrasound. Therefore, radiological findings of SBC overlap with other benign fibro-epithelial lesions of the breast. In the present case, the mammography also showed a well-defined, sub-areolar, radio-opaque lesion with no evidence of micro calcification.^5^

As mentioned previously, the SBCs are frequently non-reactive for hormone receptor analysis (ER, PR, HER2/neu) with strong and diffuse cytoplasmic positivity for S-100. Therefore, SBC commonly behaves as TNBC. Perineural and lymphovascular invasions are also extremely rare in SBC. Strong and diffuse cytoplasmic positivity for S-100 is a very common finding.^18^

The Characteristic genetic translocation (ETV6-NTRK3) of SBCs creates a fusion gene that codes a chimeric tyrosine kinase, which subsequently activates Ras-Mek1 and PI3K-Akt pathways, resulting in increased transforming and mitogenic activity of the fibroblasts and ductal epithelial cells. The chimeric protein also contributes to the expression of mammary growth factor protein in SBC, which is the key protein causing secretory changes, and its expression in SBCs is also very unique.^19^ ^,^ ^20^

The *NTRK* gene fusion involving *NTRK1, NTRK2,* or *NTRK3* is present in several tumors, like; congenital mesoblastic nephroma, infantile fibrosarcoma, mammary analog secretory carcinoma and many other tumors of different organs which make them susceptible to tyrosine kinase inhibitors (TKI).^21^ Larotrectinib and entrectinib are the first generations of TKIs. The efficacy of the larotrectinib was studied in three clinical trials. According to the published data of 55 patients, where one breast cancer patient was included, it showed an 80% overall response rate. Whereas the partial and complete response was 62% and 13%, respectively.^22^ Entrectonib was also tried on tumors harboring *NTRK* fusions. However, no breast tumor patient was included.^23^ Both the drugs were well tolerated with mild side effects. Therefore, they can be used in *TRK*-fusion positive cancers. So far, two types of resistance to first-generation TKIs are known. One is on target, and the other one is off-target resistance. Next-generation TKIs can overcome the resistance to first degree TKIs.^21^ ^,^ ^22^

The incidence of lymph node metastasis is seen in 30% of patients, and a negative sentinel lymph node biopsy is usually associated with a better prognosis. If the size of the tumor is less than 2 cm, then, the chance of metastasis is also low.^24^ Horowitz et al.^2^ reviewed the SEER database of 83 patients of SBC. According to that, 80% of the younger cohort (<30 years) were treated with a simple mastectomy, and only one patient received adjuvant radiation. Whereas 48.7% of the elderly patients (>30 years) were treated with lumpectomy, and 56% of the same cohort received radiation. Radiation therapy increases long term survival in SBC, just like other invasive breast malignancies.^25^ In our case, the affected individual is a 60-year-old lady treated with breast conservation surgery and radiotherapy. As SBC is a slowly progressing tumor with the very infrequent possibility of metastasis, response to chemotherapy is low.^25^ Herz et al.^26^ in his case study also showed non-responsiveness of SBCs to the adjuvant chemotherapy. Therefore, adjuvant chemotherapy was not considered for our patient. She is disease-free at 1 year of follow up.

## CONCLUSION

Secretory breast carcinoma is an uncommon, triple-negative breast tumor with a favorable clinical course. Radiologically, this tumor closely mimics fibroadenoma and other benign breast lesions. The morphology and genetic translocation of this tumor are also quite characteristic. There are no consensus guideline recommendations for the treatment of SBCs. Due to the indolent and slow‐growing nature of this tumor, most recurrences are seen between 10‐20 years after the initial presentation. Therefore, all the patients irrespective of their age demand long term follow-up.
